# Supplementary material for: Splice-Junction-Based Mapping of Alternative Isoforms in the Human Proteome
Source: Cell Rep. Author manuscript; Available in PMC 2020 Jan 15. (PMC6961840; doi:10.1016/j.celrep.2019.11.026)
Supplement: 2 [file NIHMS1546469-supplement-2.zip › DF1/PXD010154/Heart/Heart_10_TTN_SNCTVSVHVSEPPR.pdf]

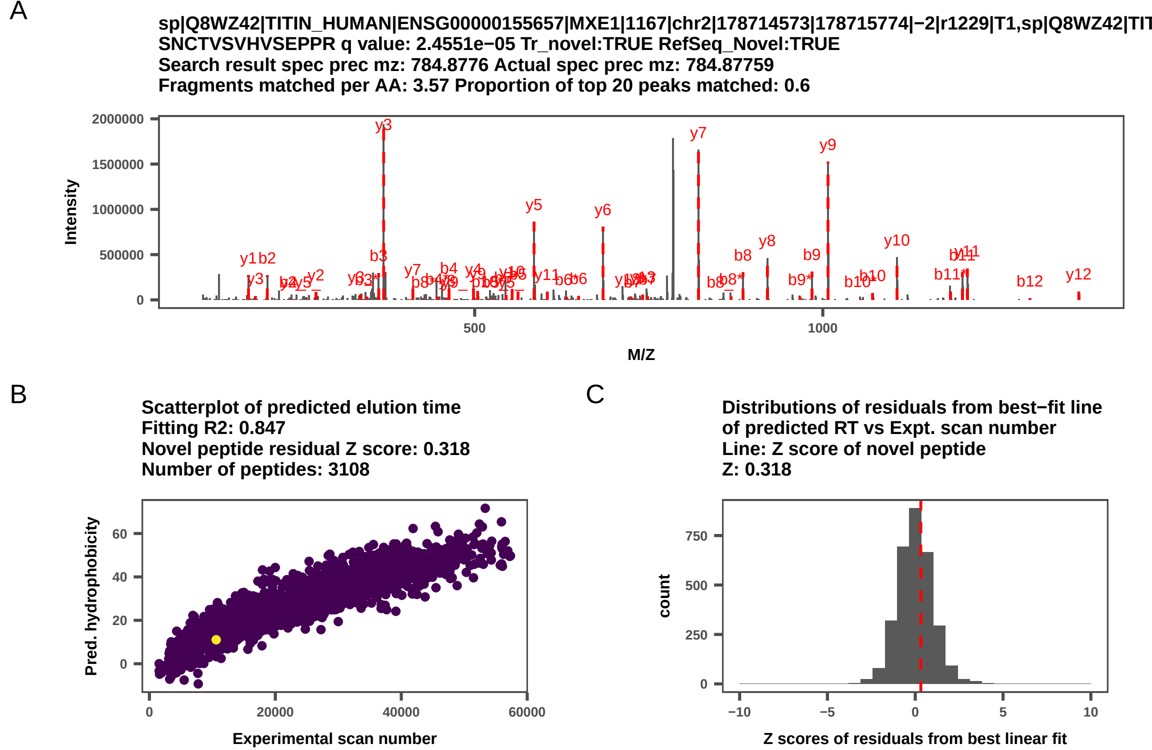
A
sp|Q8WZ42|TITIN_HUMAN|ENSG00000155657|MXE1|1167|chr2|178714573|178715774|−2|r1229|T1,sp|Q8WZ42|TIT
SNCTVSVHVSEPPR q value: 2.4551e−05 Tr_novel:TRUE RefSeq_Novel:TRUE
Search result spec prec mz: 784.8776 Actual spec prec mz: 784.87759
Fragments matched per AA: 3.57 Proportion of top 20 peaks matched: 0.6
Intensity
2000000
1500000
1000000
500000
0
M/Z
500
1000
y3
y7
y9
y5
y6
y8
b8
b9
y10
b12
y12
B
Scatterplot of predicted elution time
Fitting R2: 0.847
Novel peptide residual Z score: 0.318
Number of peptides: 3108
Pred. hydrophobicity
60
40
20
0
Experimental scan number
0
20000
40000
60000
C
Distributions of residuals from best−fit line
of predicted RT vs Expt. scan number
Line: Z score of novel peptide
Z: 0.318
count
750
500
250
0
Z scores of residuals from best linear fit
−10
−5
0
5
10
